# Beyond Reimbursement Status: Availability of Advanced Therapy Medicinal Products Across the European Union

**DOI:** 10.1007/s43441-025-00769-z

**Published:** 2025-04-10

**Authors:** Zora Čechová, Jana Kubátová, Adéla Bártová, Jakub Jamárik, Jiří Samek

**Affiliations:** 1 Centre of Excellence CREATIC, Faculty of Medicine, Masaryk University, Kamenice 5, Brno, 62500 Czech Republic; 2 Department of Pharmacology, Faculty of Medicine, Masaryk University, Brno, Czech Republic

**Keywords:** Cell therapy, Gene therapy, Patient access, Inequity, Pharmaceutical market, National competent authority

## Abstract

**Background:**

Advanced Therapy Medicinal Products (ATMPs) represent an innovative therapeutic approach with the potential to impact the treatment of rare diseases significantly. Although authorised centrally in the European Union, their market launch differs across Member States (MS). This study aimed to describe the ATMP market availability in MS and explore potential influencing factors, providing insights into specific barriers beyond pricing and reimbursement policies.

**Methods:**

ATMP availability was defined as the product launch in each MS. Data was collected through open governmental sources, databases, and communication with national competent authorities. Spearman’s correlation coefficients were calculated to examine the relationship between ATMP availability and their characteristics (time since granting marketing authorisation, target patient population size, and cost).

**Results:**

We collected the availability data on 18 ATMPs from 23 EU MS. Market uptake varied significantly, with Germany (89%), France and Italy (61%) leading. Estonia and Latvia confirmed that no ATMP has been launched on their markets yet. Six ATMPs were available in more than one-third of the analysed MS. No significant correlation was observed between ATMP availability and analysed product characteristics except for time dependency for CAR T-cell therapies.

**Conclusion:**

Beyond pricing and reimbursement processes, the ATMP commercialisation in particular MS is influenced by the marketing authorisation holder’s decision and capacity. ATMPs face product-specific challenges in achieving EU-wide availability, including complex manufacturing, distribution, and administration processes. To increase the accessibility of innovative ATMP-based treatments, implementing the cross-border access framework or individual ATMP production under the hospital exemption is essential, especially in underserved MS.

**Supplementary Information:**

The online version contains supplementary material available at 10.1007/s43441-025-00769-z.

## Introduction

The sector of Advanced Therapy Medicinal Products (ATMPs), which includes gene therapies, somatic cell therapies and tissue-engineered products, is rapidly evolving. By the end of 2023, 18 ATMPs were authorised in the European Union (EU). The majority was developed to treat rare diseases and holds the orphan designation for 21 indications [1, 2]. Over three hundred ongoing clinical trials in Europe [3], the authorisation of two new products, and the evaluation of six more [4] in the first half of 2024 show that these medicinal products offer therapeutic options for diseases where the conventional treatment remains ineffective or unavailable.

According to the European Charter of Patient’s Rights, patients in the EU Member States (EU MS) have the right to medical innovations, which is further described as *‘the right of access to innovative procedures according to international standards and independently of economic or financial considerations’* [5]. The EU supports patient access to innovative medicines, including ATMPs, through several tools, such as scientific advice and centralised marketing authorisation under conditional approval or exceptional circumstances, and with accelerated assessment. However, granting centralised marketing authorisation is only the initial step and does not guarantee accessibility across EU MS.

The proposal for new European pharmaceutical legislation underscores the interconnected nature of access depending on both availability and affordability, and recognises that some patients may not benefit from innovative medicines due to limited access. Availability is achieved only when medicine is placed on the market and manufactured in sufficient quantities to meet patients’ needs, as outlined in the European Medicines Agencies Network Strategy (EMANS) 2028 [6]. Launching an ATMP in a particular MS is dependent primarily on the intention of the marketing authorisation holder (MAH). The subsequent processes of pricing and reimbursement (P&R), which determine the affordability, then remain within the competency of an individual MS as they should align with national health needs and resources [7]. 

ATMP availability in Europe is a surprisingly understudied topic. Recent analyses focus mainly on the affordability for national healthcare systems, reimbursement processes and health technology assessment (HTA) policies, primarily covering the EU5 countries (Germany, France, Italy, Spain, and formerly the United Kingdom) [8–12]. Focus on other EU MS or the entire EU is much less frequent [13–15]. Although the publication of the HTA recommendation or reimbursement decision does not guarantee the actual availability of medicines to patients [16], the results of the studies mentioned above have already indicated important differences in the number of assessed or reimbursed products among the main EU markets and therefore, a potential access inequality in the EU. As complex research on the actual market availability of ATMPs EU-wide is lacking, our study focuses on this availability rather than affordability and contributes to a comprehensive description of patient access to these products.

Our study aims to (1) describe the market availability of all authorised ATMPs in EU MS; (2) explore the potential influencing factors such as the time since granting marketing authorisation, target patient population size, and cost; and (3) discuss the availability in the context of the unique properties of ATMPs, beyond P&R policies.

## Methods

This study analyses the market availability of authorised ATMPs defined by Regulation (EC) No 1394/2007 and Directive 2001/83/EC [17, 18] in the EU MS markets. ATMP was considered to be available if its market launch in the MS occurred from the date of marketing authorisation until March 5, 2024 (data lock point of this study).

### Data Extraction and Verification

The list of the ATMPs, including information on the orphan designation and marketing authorisation date, was extracted from the Committee for Advanced Therapies meeting reports [2]. The time since granting marketing authorisation was calculated as the difference between the marketing authorisation date and March 2024, counting all commenced years.

The average annual cost of each ATMP (hereafter referred to as “cost”) and the number of eligible patients (hereafter referred to as “target patient population size”) were retrieved from the benefit assessments published by the German Federal Joint Committee (G-BA) [19]. Using data from a single MS served to explore the differences among individual products. HTA reports from Denmark, France, Germany, the Netherlands, and the United Kingdom were considered. We finally selected G-BA data based on their completeness and availability for the largest number of ATMPs (15 out of 18 for target population size, 14 out of 18 for cost).

Sources of information and indicators of ATMPs availability were established for each MS. Firstly, the official “List of Reference Sources” of the State Institute for Drug Control (Czech national competent authority, NCA) [20] was screened as a source of verified information. The Czech NCA uses this list to ascertain the market availability of assessed medicinal products in P&R processes. Secondly, a comprehensive search of the institutional websites of NCAs and other government institutions was conducted to obtain details on marketing/commercialisation status, first launch, distribution, and dispensation data.

The content of these national databases or lists was inconsistent regarding the scope and types of information included. Some sources contained all authorised medicinal products, while others only listed those that are marketed or fulfil the conditions to be marketed. Several sources lacked information on actual availability of medicinal products (Austria, Cyprus, Denmark, Greece, the Netherlands, Slovenia), availability of centrally authorised medicinal products (Ireland), or if availability was indicated, the term had unclear meaning. In contrast, some MS publish detailed distribution or dispensation data (Czech Republic, Slovakia, Lithuania) in which the actual availability status is clear.

Described inconsistencies were addressed through direct communication with the NCAs. We verified whether the sources reliably indicated market launch and asked the NCAs to confirm the particular ATMP launch. Email communication with NCAs was the sole source of information for Bulgaria, Poland, and Italy. The list of all sources investigated is given in Supplementary Table 1. Supplementary Table 2 presents the final sources of information and indicators of market availability for EU MS, including information about verification by NCAs.

### Data Analysis

ATMP availability was first analysed using descriptive statistics. We calculated the number and ratio of market-launched ATMPs to authorised ATMPs available in each MS and the number of MS where each product was available.

Correlation analysis assessed the relationship between the number of MS where each ATMP was available and the following product characteristics: time since marketing authorisation was granted, target patient population size, and cost. Analysis was performed for all ATMPs for which data were available. Subgroup analysis was performed for gene therapy medicinal products (GTMP), somatic cell therapy medicinal products (sCTMP), tissue-engineered products (TEP), autologous products, non-autologous products (i.e., products not using autologous material for the manufacture), and CAR T-cell therapies. The normality of each variable was assessed visually using histograms and Q-Q diagrams. As normality could not be assumed, Spearman’s correlation coefficient was computed for each pair. Analysis was performed using R version 4.3.2.

## Results

By the end of 2023, 18 ATMPs had been authorised in the EU. The availability data were obtained for 23 out of 27 EU MS: Belgium, Bulgaria, Croatia, Cyprus, Czech Republic, Denmark, Estonia, Finland, France, Germany, Greece, Italy, Latvia, Lithuania, Luxembourg, the Netherlands, Poland, Portugal, Romania, Slovakia, Slovenia, Spain, and Sweden. Four MS were excluded from this study: Austria and Ireland, where NCAs confirmed that data are unknown; and Hungary and Malta, where no ATMPs were indicated as available, and the NCAs did not confirm this finding. The NCAs provided verification for 20/27 EU MS (74%; Supplementary Table 2).

**Fig. 1:**
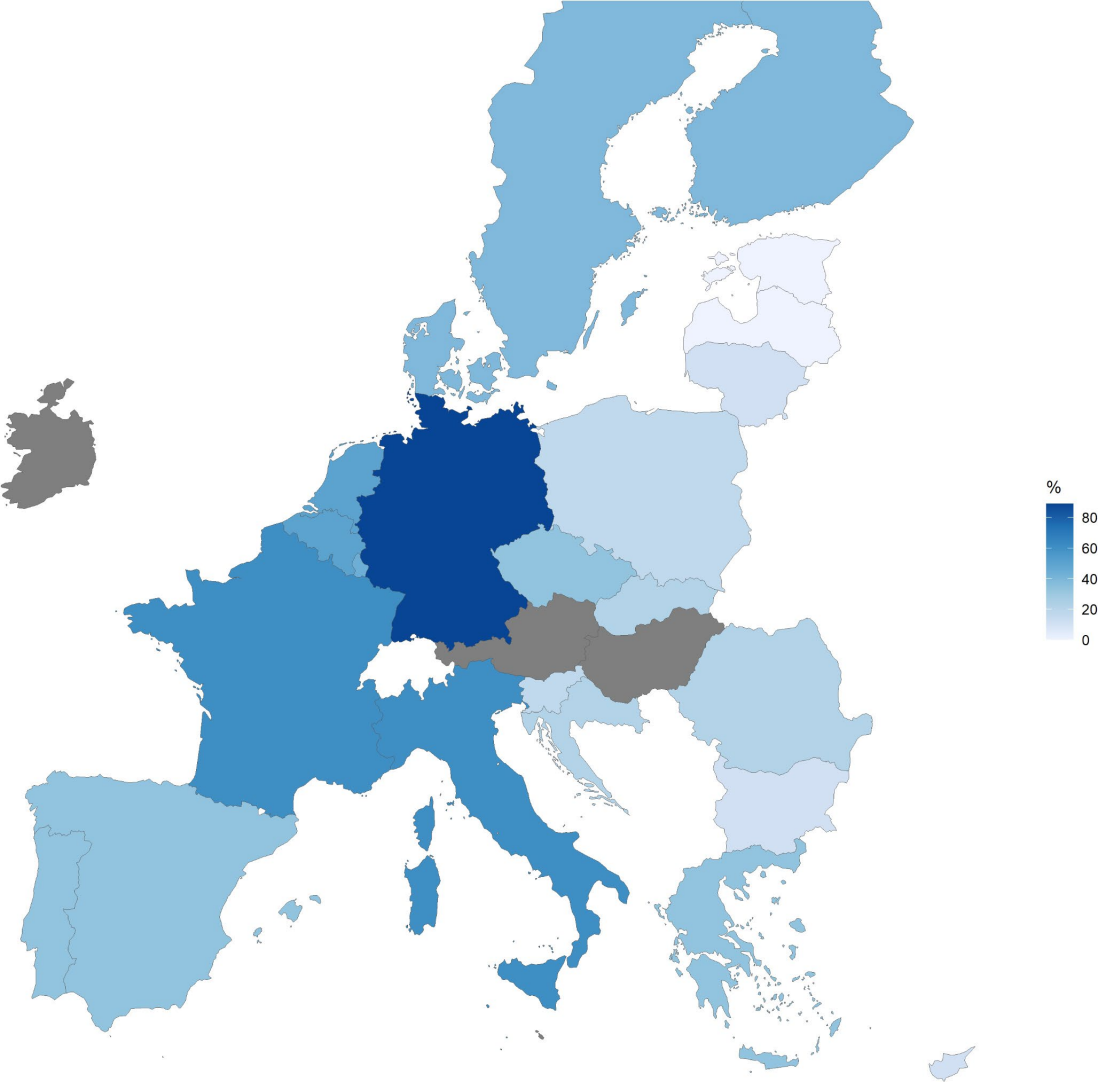
Geographical distribution of ATMP availability across EU Member States expressed as a percentage of authorised products. Grey areas indicate EU MS excluded from the analysis. Abbreviations: ATMP, advanced therapy medicinal product

Figure 1 presents a heat map illustrating the geographical distribution of ATMP availability in EU MS. The highest ratio of ATMP availability was found in Germany (89%), followed by France and Italy (both 61%). No ATMPs have been launched in Estonia and Latvia yet. The availability ratio is lower than the average of analysed EU MS (26%) in 10/23 evaluated MS. Detailed data are provided in Supplementary Tables 3 and 4.

Table 1 provides a detailed overview of the ATMPs ranked by the number of EU MS where they are available, including information on the indication, orphan designation status, year of marketing authorisation, ATMP cost, and size of the target patient population. Six ATMPs were available in more than one-third of the analysed MS. Notably, three of them are CAR T-cell therapies used in haematologic malignancies and have multiple indications (Kymriah, Tecartus, and Yescarta); four were authorised more than five years ago (Alofisel, Kymriah, Luxturna, and Yescarta).

**Table 1:** Overview of medicinal product characteristics. ATMPs are arranged by the number of EU MS in which they are available and the bold line separates six products available in more than one-third of the analysed EU MS

ATMP	Number of EU MS (% of analysed MS)	Indication	Orphan designation	Year of MA	Cost (EUR)^1^	Size of the target patient population (*n*)^2^
Zolgensma	19 (83%)	spinal muscular atrophy	Y	2020	2,314,550	45–65
Kymriah	18 (78%)	follicular lymphoma, diffuse large B-cell lymphoma, B-cell acute lymphoblastic leukaemia	Y	2018	239,000	1,220–1,980
Alofisel	16 (70%)	perianal fistulas in patients with Crohn’s disease	Y	2018	71,400	90–230
Yescarta	15 (65%)	high-grade B-cell lymphoma, diffuse large B-cell lymphoma, primary mediastinal large B-cell lymphoma, follicular lymphoma	Y	2018	272,000	760–1,470
Luxturna	13 (57%)	inherited retinal dystrophy	Y	2018	702,100	100–530
Tecartus	11 (48%)	acute lymphoblastic leukaemia, mantle cell lymphoma	Y	2020	282,000	186–350
Ebvallo	5 (22%)	Epstein-Barr virus-positive post-transplant lymphoproliferative disease	Y	2022	535,000–2,142,000	7–30
Holoclar	5 (22%)	limbal stem-cell deficiency	Y	2015	ND	ND
Imlygic	5 (22%)	unresectable melanoma	N	2015	72,287.8–289,151.2	375–670
Libmeldy	5 (22%)	metachromatic leukodystrophy	Y	2020	2,875,000	1–3
Spherox	4 (17%)	symptomatic articular cartilage defects of the femoral condyle and the patella of the knee	N	2017	ND	ND
Upstaza	4 (17%)	severe aromatic L-amino acid decarboxylase deficiency	Y	2022	4,165,000	4–30
Abecma	3 (13%)	multiple myeloma	Y	2021	350,000	1,200–1,300
Breyanzi	3 (13%)	diffuse large B-cell lymphoma, high-grade B-cell lymphoma, primary mediastinal large B-cell lymphoma, follicular lymphoma grade 3B	N	2022	345,000	835–1,180
Roctavian	3 (13%)	haemophilia A	Y	2022	2,143,958.4	690–800
Hemgenix	2 (9%)	haemophilia B	Y	2023	ND	340
Carvykti	1 (4%)	multiple myeloma	Y	2022	420,000	1,210–1,310
Strimvelis	1 (4%)	severe combined immunodeficiency due to adenosine deaminase deficiency	Y	2016	ND^3^	ND

Abbreviations: ATMP, advanced therapy medicinal product; MA, marketing authorisation; MS, Member States; N, no; ND, no data; Y, yes

Notes: ^1^ average annual cost in Germany [19], ^2^ number of eligible patients in Germany [19], ^3^ ATMP cost in Italy: 594,000 EUR [41]

**Fig. 2:**
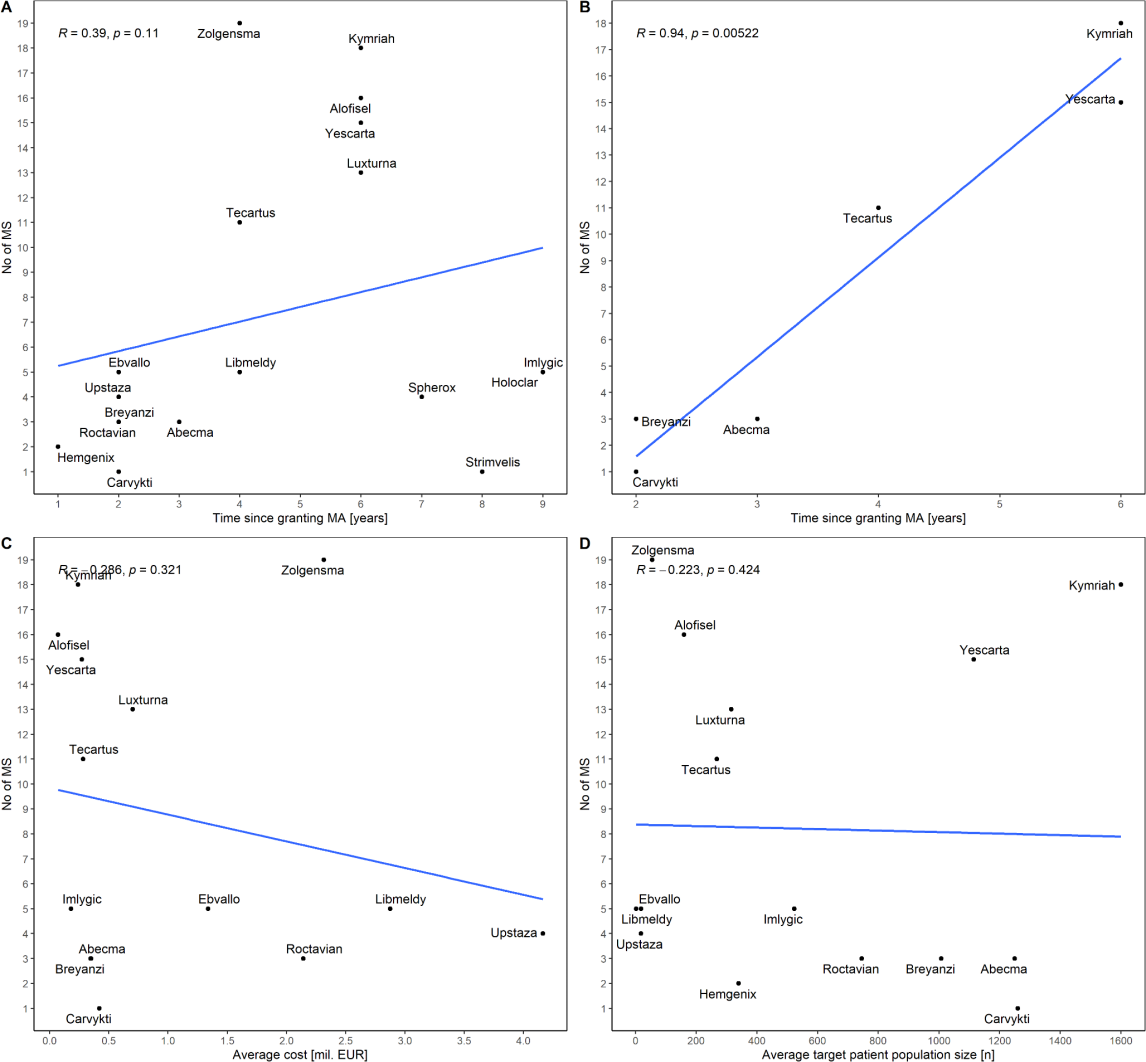
Relation between the ATMP availability in the EU Member States and (**A**) time since granting marketing authorisation, (**B**) time since granting marketing authorisation for CAR T-cell therapies, (**C**) cost, (**D**) size of the target patient population. Abbreviations: ATMP, advanced therapy medicinal product; MA, marketing authorisation; MS, Member States

No statistically significant correlation (*p* > 0.05) was observed between the number of MS where ATMP is available and the following factors: time since granting marketing authorisation (*n* = 18, Fig. 2A), target patient population size (*n* = 15, Fig. 2D), and cost (*n* = 14, Fig. 2C). In subgroup analysis, a statistically significant correlation (*p* < 0.05) was found between the number of MS and: (a) time since granting marketing authorisation for CAR T-cell therapies (Fig. 2B), (b) cost of CAR T-cell therapies (Supplementary Fig. 3B), (c) cost of autologous products (Supplementary Fig. 2B). The correlation between the number of MS and the cost of autologous products was driven by an outlier observation, indicating a statistical artefact. Due to insufficient sample sizes, the analysis was not feasible for sCTMP (*n* = 2) and TEP (*n* = 2). All figures for subgroup analyses are available in the Supplementary file (Supplementary Figs. 1–3).

## Discussion

### Methodological Aspects of Determining ATMP Availability

Our study describes the market availability of authorised ATMPs across 23 EU MS, addressing a regional gap in previous research in which Central and Eastern EU MS were underrepresented. The main difference in our methodology lies in the definition of ATMP availability. Previous studies analysed the situation based on reimbursement status [8–10, 14, 21]. That reflects affordability but may underestimate availability, as these products may reach patients even in the absence of a formal reimbursement decision.

This discrepancy is highlighted when comparing our results with a recent report published by the Danish Association of the Pharmaceutical Industry (Lif). Lif mapped ATMP access in 11 European countries based on the information provided by local industry associations [14]. The report aligns with our findings only in Germany, where statutory health insurance funds automatically cover newly marketed products. In all other countries, the Lif report identified lower availability than in our study. A significant difference was observed in Italy (five available ATMPs compared to eleven in our study). Italian regulatory framework provides immediate availability of innovative medicinal products to patients through inclusion in class C(nn), even without formal inclusion in the regional hospital therapeutic schedules [22]. 

ATMPs can also be accessed on a case-by-case basis following the special requests [21]. For instance, in Bulgaria, patients can access two ATMPs after a request by a medical specialist, followed by an eligibility check by the Ministry of Health and Bulgarian Drug Agency. Additionally, discrepancies may arise from differing reimbursement processes for inpatient and outpatient settings. In the Czech Republic, only two of the six marketed ATMPs underwent the formal reimbursement procedure, which applies only to medicinal products in outpatient settings, a criterion that most ATMPs do not meet.

Sales data may serve as another source for evaluating market availability [23, 24]. However, such data are typically not fully accessible to the public. Our study relies on open or requestable data, although their inconsistency presents a primary limitation complicating the accurate determination of market availability. While both sales data and our study data reflect only one aspect of patient access – availability – they should not be neglected. Standardising data collection and reporting practices by NCAs would enhance future research accuracy and offer a more comprehensive understanding of how ATMPs reach patients.

### Variability of ATMP Availability across EU MS

We observed significant variation in the ATMP availability among EU MS, ranging from 0 to 89%. Factors leading to unequal availability may parallel those observed for orphan medicinal products: national health strategies for rare diseases, diverse national P&R policies, and economic aspects, such as public health resources and market size [16, 23–25].

Germany exhibited the highest ratio of ATMPs marketed, which aligns with previous studies confirming its leadership in the availability of centrally authorised products, orphan medicinal products, and fast patient access [16, 23, 26]. This position derives from Germany’s policy allowing reimbursement and free price setting for all new treatments during the first six months [13] and its inclusion in the reference pricing systems of other countries. Similarly, legislative measures in Italy, through the “innovative product” designation, and in France, through the “Temporary Authorisation for Use” (ATU) program, foster patient access to innovative therapies addressing unmet medical needs [27]. Germany, France, and Italy, as MS with the highest ATMP availability in this study, emphasise how market size, innovation-supporting policies, and reimbursement outcomes of previously launched ATMPs shape commercial strategies by MAHs.

In contrast, no ATMPs have been marketed in Estonia and Latvia to date of research, which is consistent with EFPIA (European Federation of Pharmaceutical Industries and Associations) surveys (based on the reimbursement status) reporting low availability rates of centrally authorised products and orphan medicinal products in the Baltic States. These surveys also identified Malta as the EU MS with the lowest availability rate; however, this MS was not included in our study as NCA did not confirm that no ATMP is available [26, 28]. Smaller markets usually encounter delays in the launch of new medicines [29]. Securing timely access to these treatments often necessitates implementing cross-border healthcare solutions.

However, market size alone does not guarantee the availability. In Spain, despite being included in the main EU4 markets, the availability of ATMPs is 33% (six ATMPs), slightly above the average of analysed EU MS. While MAHs express the intention to commercialise 83% of ATMPs (15/18) in Spain, no agreements on P&R conditions have been reached for four ATMPs, and the discussions for the remaining are ongoing [30]. The willingness of national health systems to pay for ATMPs is affected by the high short-term cost of ATMPs combined with limited efficacy data and uncertainty in long-term benefits [31]. The decision-making in this field is complex and shaped by various clinical and economic considerations and price agreements, as addressed comprehensively in recent literature [10, 12, 13, 32, 33]. An important step in this context is the recent EU Health Technology Assessment Regulation (Regulation (EU) 2021/2282), which mandates Joint Clinical Assessments (JCA) for newly authorised oncology medicinal products and ATMPs from January 2025 [34]. The single scientific basis for national decisions is expected to facilitate patient access to innovative treatments across the EU. However, challenges in adapting national HTA processes to integrate the JCA findings effectively may paradoxically delay market access in the initial phase.

Beyond market dynamics and regulatory environment, ATMP availability is further influenced by product-specific limitations in manufacture, distribution, and administration, which MAH has to deal with. The manufacturing complexity is not comparable to other biological medicinal products and may strain the manufacturing capacity. “Fresh products” with limited shelf-life require specialised handling, temperature chain maintenance, and fast administration by trained healthcare professionals in specialised facilities certified by MAHs [35]. Additionally, some countries may face organisational and technical issues in establishing centres suitable for administering ATMPs [36]. Countries involved in ATMP clinical trials may be preferred for initial product launches. Their prior experience with product administration and established supply chains may provide a strategic advantage in facilitating early access. This aligns with our findings, as most ATMP clinical trials in the EU are conducted in France, Germany, Italy and Spain, followed by Belgium and the Netherlands [37]. 

All these regulatory or product-specific limitations may lead to difficulties in commercialisation, prioritisation of MS and unequal availability. Larger, more established companies can generally expand across a wide range of markets more quickly than small-medium enterprises [38]. 

### Analysed Factors Influencing ATMP Availability

Our study further explores the potential influence of three factors on ATMP availability across EU MS, covering the practical, commercial and economic dimensions relevant to the market access: time since granting marketing authorisation, target patient population size, and cost. We found no significant correlation between the number of MS where an ATMP is available and the analysed factors when evaluating all ATMPs. These factors do not relate to overall ATMP availability linearly, suggesting a more complex decision process by all involved stakeholders and emphasising the specific nature of each ATMP. The correlation analysis is further complicated by a limited number of authorised ATMPs and a short period since marketing authorisations.

When analysing ATMP subgroups, time dependency can be observed for CAR T-cell therapies. This finding suggests that recently authorised products may require more time to build a sufficient infrastructure, leading to delayed launches in multiple countries. Time is crucial for manufacturing scale-up and establishing distribution networks and certified facilities across MS. CAR T-cell therapies and other autologous products may require a more robust network, as the treatment process involves the collection of patients’ cells and their transport for product manufacturing. This time dependency does not apply to all ATMPs. Although four of the six most available ATMPs were authorised for over five years at the time of research, there are ATMPs authorised for a longer time with lower availability. That points to the interconnection of various factors influencing the ATMP expansion to multiple MS.

We also observed a correlation between the availability of CAR T-cell therapies and cost; however, this finding requires cautious interpretation. Given the data source, Germany, with its unique market position, and the existing price differences across EU MS, this study should provide rather an exploratory insight than definitive conclusions applicable to all EU MS. Additionally, the final cost of medicines is shaped by managed entry agreements (MEA) and confidential pricing agreements, which facilitate negotiations between MAHs and healthcare payers and determine patient access. The list prices of CAR T-cell therapies are remarkably similar, making cost comparisons within this category irrelevant. Rather than focus on the exact costs of individual products, we can consider the cost range from €71,400 to over €2,3 mil for the six most available products to illustrate the lack of cost impact. Notably, the costliest product is also the most available (present in 83% of analysed EU MS), highlighting that although the cost and P&R negotiations influence the final patient access, it is not the only factor that drives ATMP availability.

Our study did not confirm any relationship between ATMP availability and the size of the target population. Two opposing mechanisms may contribute to this finding. On the one hand, greater demand from patients and healthcare professionals is expected to facilitate ATMP availability but simultaneously creates challenges for manufacturing capacity. This constraint might be expected for autologous products, such as CAR T-cell therapies, but it is also documented for an allogenic product, Alofisel. The Danish Nordic Institute of Health Economics reported that the number of eligible patients for Alofisel treatment was between 540 and 555 at the time of application submission for HTA evaluation. Nevertheless, the manufacturing capacity allows for treating only 20 to 45 patients annually [39]. 

On the other hand, extremely small patient populations present a distinct challenge, especially when combined with distribution and administration requirements, as illustrated by Strimvelis and Upstaza. Strimvelis has been administered exclusively in one facility since 2016, as only 15 patients per year are estimated to be eligible in Europe. The administration and manufacturing sites are co-located, ensuring fast administration due to the limited shelf-life of several hours. Instead of expanding into additional MS markets, the MAH promotes cross-border access to treatment by establishing a patient support program [40, 41]. Upstaza requires administration in centres specialised in stereotactic neurosurgery by qualified neurosurgeons [42]. This requirement, combined with a small target population (only units of patients), concentrates the product in a few treatment centres across Europe.

## Conclusion

Our study identified significant variation in ATMP availability across EU MS with no evident relation between availability and factors such as time since granting marketing authorisation, target population size, and cost when analysing all ATMPs together. This suggests that decision-making processes are complex and specific to each ATMP. Although diverse national P&R processes are often highlighted as the main obstacles for MAHs, product-specific limitations in manufacture, distribution, and administration cannot be omitted when discussing EU-wide availability. All these challenges lead MAHs to prioritise certain MS for product launches, a decision understandable from a business perspective. However, this prioritisation negatively impacts fair access to innovative treatments, mainly affecting patients without alternative treatment options.

Greater transparency is needed to improve patient access to innovative ATMP-based treatments. NCAs could enhance this by reporting the availability status of ATMPs, providing deeper insight into patient access patterns. Likewise, more transparent communication from MAHs regarding market entry timelines/decisions would support the preparedness of national healthcare systems to adopt these products in clinical practice. The underserved MS should consider alternative approaches to secure the medical needs, such as implementing the cross-border access frameworks or individual ATMP production under the hospital exemption. Addressing these challenges requires coordinated efforts from regulatory bodies on the European and national levels, healthcare providers, and pharmaceutical companies to enhance patient access and optimise the benefits of ATMPs.

Future research should focus on longitudinal analysis to monitor time trends and regional disparities in ATMP availability across EU MS and assess the impact of the EU HTA Regulation on ATMP access.

## Electronic Supplementary Material

Below is the link to the electronic supplementary material.

Supplementary Material 1

## Data Availability

No datasets were generated or analysed during the current study.
