# Supplementary material for: Beyond Reimbursement Status: Availability of Advanced Therapy Medicinal Products Across the European Union
Source: Ther Innov Regul Sci. 2025 Apr 10;59(4):728–36. doi: 10.1007/s43441-025-00769-z (PMC12181088; doi:10.1007/s43441-025-00769-z)
Supplement: Supplementary file 1 — Supplementary Material 1 [file 43441_2025_769_MOESM1_ESM.docx]

**Supplementary Material**

1. **Supplementary Tables**

**Supplementary Table 1.** List of all sources investigated.

| EU Member State | Competent agency | Reference name |
| --- | --- | --- |
| Austria | Austrian Agency for Health and Food Safety (AGES) | Email communication |
|  | Austrian Federal Office for Safety in Health Care (BASG) | Email communication |
|  |  | Arzneispezialitätenregister [1] |
|  | Gesundheit Österreich | Email communication |
|  | Österreichische Sozailversicherung | Erstattungskodex [2] |
| Belgium | Federal agency for medicines and health products (FAMHP) | Email communication |
|  |  | PharmaStatus [3] |
|  | Rijksinstituut voor ziekte- en invaliditeitsverzekering (RIZIV) | Vergoedbare geneesmiddelen en radio-farmaceutische producten [4] |
| Bulgaria | Bulgarian Drug Agency | Email communication |
|  |  | Register of pharmaceutical products [5] |
|  | National Council on Prices and Reimbursement of Medicinal Products (NCPR) | Registers of National Council on Prices and Reimbursement of Medicinal Products [6] |
|  | National Health Insurance Fund | Списъци с лекарствени продукти [7] |
| Croatia | Croatian Agency for Medicinal Products and Medical Devices (HALMED) | Email communication |
|  |  | Baza lijekova/Medicinal Product Database [8] |
|  | Croatian Health Insurance Fund | Osnovna lista lijekova [9] |
| Cyprus | Pharmaceutical Services Ministry of Health | Email communication |
|  |  | Price list [10] |
|  |  | Product search [11] |
| Czech Republic | State Institute for Drug Control (SÚKL) | Email communication |
|  |  | Opatření obecné povahy [12] |
|  |  | Přehled správních řízení [13] |
|  |  | Otevřená data: REG-13; DIS-13; LEK-13; Dodávky léčivých přípravků [14] |
| Denmark | Danish Medicine Agency | Email communication |
|  |  | Medicinpriser.dk [15] |
|  | Danish Medicines Council | Medicinrådet: Anbefalinger og vejledninger [16] |
| Estonia | State Agency of Medicines (SAM) | Email communication |
|  |  | Register of Medicinal Products [17] |
|  | Tervisekassa – Health Insurance Fund | Soodusravimid [18] |
| Finland | Fimea – Finnish Medicines Agency | Email communication |
|  |  | FimeaWeb [19] |
|  | Kela | Medicinal Products Database [20] |
| France | French National Agency for Medicines and Health Products Safety (ANSM) | Email communication |
|  |  | Répertoire des Spécialités Pharmaceutiques [21] |
|  | French National Authority for Health (HAS) | Avis et décisions sur les medicaments [22] |
|  | Ministry of Health | Référentiel des indications des spécialités pharmaceutiques inscrites sur la liste en sus [23] |
| Germany | Gemeinsamet Bundesausschuss (G-BA) | Nutzenbewertung von Arzneimitteln [24] |
|  | Paul-Ehrlich-Institute | Email communication |
|  | CGM Lauer | Lauer-Taxe [25] |
| Greece | Ministry of Health | Φάρμακα Υψηλού Κόστους – καταλόγων για τη θεραπεία σοβαρών ασθενειών [26] |
|  | National Organization for Medicines | Email communication |
|  |  | Search human product [27] |
| Hungary | National Center for Public Health and Pharmacy | Email communication |
|  |  | Gyógyszer-adatbázis [28] |
|  | National Health Insurance Fund of Hungary (NEAK) | Publikus Gyógyszertörzs (PUPHA) – Végleges [29] |
| Ireland | Health Products Regulatory Authority (HPRA) | Email communication |
|  |  | Find a medicine [30] |
|  | Health Service Executive | Reimbursable Items [31] |
|  | National Centre for Pharmacoeconomics (NCPE) | Assessments of National Centre for Pharmacoeconomics [32] |
| Italy | Italian Medicines Agency (AIFA) | List of Class H medicinal products [33] |
|  |  | Innovative drug list [34] |
|  |  | List of Class C(nn) medicinal products [35] |
|  | Farmadati Italia | Email communication |
| Latvia | National Health Service | Assessment of applications for inclusion of new medicinal products in the list of reimbursable medicinal products [36] |
|  |  | Lists of reimbursed medicines [37] |
|  | State Agency of Medicines Republic of Latvia | Email communication |
|  |  | Medicinal product register of Latvia [38] |
| Lithuania | State Medicines Control Agency of Lithuania | Email communication |
|  |  | Parduotų vaistinėms ir ASPĮ vaistinių preparatų pakuočių kiekis [39] |
|  | National Health Insurance Fund under the Ministry of Health | Kompensuojamieji vaistai ir medicinos pagalbos priemonės [40] |
|  |  | Vaistų ir medicinos pagalbos priemonių kainų paieška [41] |
| Luxembourg | Ministère de la Santé et de la Sécurité sociale, Division de la pharmacie et des médicaments | Email communication |
|  | CNS | Liste des médicaments commercialisés [42] |
| Malta | Malta Medicines Authority | Email communication |
|  |  | Medicines Authority Advanced Search [43] |
|  | Directorate for Pharmaceutical Affairs | Government Formulary List [44] |
| Netherlands | Zorginstituut Nederland | Email communication |
|  |  | Horizonscan geneesmiddelen [45] |
|  |  | Medicijnkosten.nl > Zoeken [46] |
|  |  | Overzicht geneesmiddelen in de sluis [47] |
|  | Medicines Evaluation Board | Email communication |
|  |  | Medicines Information Bank [48] |
| Poland | Chief Pharmaceutical Inspectorate | Email communication |
|  |  | Zintegrowany System Monitorowania Obrotu Produktami Leczniczymi [49] |
|  | Ktomalek.pl | KtoMaLek.pl [50] |
|  | Ministry of Health | Leki refundowane [51] |
|  |  | Rejestr Produktów Leczniczych [52] |
|  | Office For Registration of Medicinal Products,  Medical Devices and Biocidal Products | Email communication |
| Portugal | Infarmed | Email communication |
|  |  | INFOMED Base de dados de medicamentos de uso humano [53] |
|  |  | Relatórios de avaliação de financiamento público [54] |
| Romania | Casa Națională de Asigurări de Sănătate | Lista interactivă a medicamentelor [55] |
|  | Ministry of Health | Catalogul Public național al prețurilor maximale ale medicamentelor de uz uman [56] |
|  | National Agency for Medicines and Medical Devices in Romania (ANMDMR) | Email communication |
|  |  | Lista medicamentelor din NOMENCLATOR [57] |
| Slovakia | Ministry of Health | Zoznam kategorizovaných liekov [58] |
|  |  | Zoznam liekov s úradne určenou cenou [59] |
|  | National Health Information Centre | Datasety spotreby humánnych liekov v Slovenskej Republike [60] |
|  | State Institute for Drug Control | Email communication |
|  |  | Oznámenie o prvom uvedení, prerušení, obnovení alebo zrušení dodávok humánneho lieku [61] |
| Slovenia | Agency for Medicinal Products and Medical Devices of the Republic of Slovenia (JAZMP) | Advanced medicinal products (ATMP) / Medicinal products authorized in Europe by now [62] |
|  |  | Email communication |
|  |  | List of regulated prices [63] |
|  | Zavod za zdravstveno zavarovanje Slovenije (ZZZS) | Podatki o porabi zdravil [64] |
|  | JAZMP + ZZZS + Ministry of Health + National Institute of Health (NIJZ) | Centralna baza zdravil [65] |
| Spain | Ministry of Health | BIFIMED: Buscador de la Información sobre la situación de financiación de los medicamentos [66] |
|  | Spanish Agency of Medicines and Medical Devices (AEMPS) | Email communication |
|  |  | CIMA - Centro de información de medicamentos [67] |
| Sweden | E-hälsomyndigheten | Email communication |
|  |  | VARA [68] |
|  | Läkemedelsindustriföreningens Service AB | FASS Allmänhet [69] |
|  | Swedish Medical Products Agency | Email communication |
|  | Tandvårds- och läkemedelsförmånsverket (TVL) | Sök priser och beslut i database [70] |
| EU | European Medicines Agency | AskEMA |

**Supplementary Table 2.** List of sources and indicators of market availability for EU Member States.

| EU Member State | Reference name | Indicator of availability | Verified by NCA |
| --- | --- | --- | --- |
| Austria^1^ | NA | NA | Y |
| Belgium | PharmaStatus [3] | available in the database | Y |
| Bulgaria | email communication (Bulgarian Drug Agency) |  | Y |
| Croatia | Baza lijekova (Medicinal Product Database) [8] | available in the database with Marketing status: “stavljeno u promet” | Y |
| Cyprus | Pharmaceutical Services Ministry of Health – product search [11] | available in the database | N |
| Czech Republic | DIS-13; LEK-13; Dodávky léčivých přípravků [14] | available at least in one of the files | Y |
| Denmark | Medicinpriser.dk [15] | available in the database | N |
| Estonia | Register of Medicinal Products [17] | available in the database with the date of last import | Y |
| Finland | FimeaWeb [19] | Marketed status: "Marketed" | N |
| France | Répertoire des Spécialités Pharmaceutiques [21] | Commercialisation: "Commercialisée" | Y |
| Germany^2^ | Lauer-Taxe [25] | not available if filed "KENNZ." contains "AV", "RW", "VP", "nw", "N", "Z" | N |
| Greece | Search human product [27] | available in the database with price | N |
| Hungary^3^ | Publikus Gyógyszertörzs (PUPHA) – Végleges [29] | table GYOGYSZ, the "FORGALOMBA" field is "1" | N |
| Ireland^4^ | NA | NA | Y |
| Italy | email communication (Farmadati Itali) |  | Y |
| Latvia | Medicinal product register of Latvia [38] | available in the database | Y |
| Lithuania | Parduotų vaistinėms ir ASPĮ vaistinių preparatų pakuočių kiekis [39] | available at least in one of the files | Y |
| Luxembourg | Liste des médicaments commercialisés [42] | available in the file | Y |
| Malta^5^ | Medicines Authority Advanced Search [43] | available in the database | N |
| Netherlands | Medicijnkosten.nl > Zoeken [46] | available in the database | Y |
| Poland | email communication (Chief Pharmaceutical Inspectorate) |  | Y |
| Portugal | INFOMED Base de dados de medicamentos de uso humano [53] | Comercialização: "Existe, pelo menos, uma apresentação comercializada." | Y |
| Romania^6^ | Lista medicamentelor din NOMENCLATOR [57] | available in the database | Y |
| Slovakia | Datasety spotreby humánnych liekov v Slovenskej Republike [60] | available at least in one of the files | Y |
| Slovenia | Centralna baza zdravil [65] | available in the database with price information | Y |
| Spain | CIMA - Centro de información de medicamentos [67] | available in the database with status "COMERCIALIZADO" | Y |
| Sweden | FASS Allmänhet [69] | information about the package is not strikethrough and the "Tillhandahålls ej" is not in the description | Y |

Abbreviations: ATMP, advanced therapy medicinal product; NA, not available; NCA, national competent authority; N, no; Y, yes

Notes:

^1^ Austrian Agency for Health and Food Safety (AGES) and Austrian Federal Office for Safety in Health Care (BASG) do not have information about the commercialisation status of centrally authorised products.

^2^ Paul-Ehrlich-Institut does not have information about the commercialisation status of centrally authorised products. The commercial source was used.

^3^ No ATMP was found. Findings were not verified by NCA.

^4^ Health Products Regulatory Authority (HPRA) does not have information about the commercialisation status of centrally authorised products.

^5^ No ATMP was found. Findings were not verified by NCA.

^6^ Alongside email communication. The database includes medicinal products for which marketing authorisation holder notifies the intention to market in Romania (Carvykti – not marketed yet).

**Supplementary Table 3.** The number and ratio of ATMPs availability (market launched to authorised ATMPs) in EU Member States (MS) as of March 5, 2024.

| EU Member State | Number of ATMPs available | Availability ratio (%) |
| --- | --- | --- |
| Austria | NA | NA |
| Belgium | 9 | 50 |
| Bulgaria | 2 | 11 |
| Croatia | 4 | 22 |
| Cyprus | 2 | 11 |
| Czech Republic | 6 | 33 |
| Denmark | 7 | 39 |
| Estonia | 0 | 0 |
| Finland | 7 | 39 |
| France | 11 | 61 |
| Germany | 16 | 89 |
| Greece | 6 | 33 |
| Hungary | NC | NC |
| Ireland | NA | NA |
| Italy | 11 | 61 |
| Latvia | 0 | 0 |
| Lithuania | 2 | 11 |
| Luxembourg | 8 | 44 |
| Malta | NC | NC |
| Netherlands | 9 | 50 |
| Poland | 3 | 17 |
| Portugal | 6 | 33 |
| Romania | 4 | 22 |
| Slovakia | 4 | 22 |
| Slovenia | 3 | 17 |
| Spain | 6 | 33 |
| Sweden | 7 | 39 |
| EU average^1^ | 4.8 | 26 |

Abbreviations: ATMP, advanced therapy medicinal product; MS, Member State; NA, not available; NC, not confirmed

Notes:

^1^ Average calculated for investigated EU MS.

**Supplementary Table 4.** ATMPs market availability in EU Member States (MS) as of March 5, 2024 according to the indicators defined in Supplementary Table 2 (green – ATMP available; grey – MS was excluded from the study).

| EU Member State | Abecma | Alofisel | Breyanzi | Carvykti | Ebvallo | Hemgenix | Holoclar | Imlygic | Kymriah | Libmeldy | Luxturna | Roctavian | Spherox | Strimvelis | Tecartus | Upstaza | Yescarta | Zolgensma |
| --- | --- | --- | --- | --- | --- | --- | --- | --- | --- | --- | --- | --- | --- | --- | --- | --- | --- | --- |
| Austria |  |  |  |  |  |  |  |  |  |  |  |  |  |  |  |  |  |  |
| Belgium |  |  |  |  |  |  |  |  |  |  |  |  |  |  |  |  |  |  |
| Bulgaria |  |  |  |  |  |  |  |  |  |  |  |  |  |  |  |  |  |  |
| Croatia |  |  |  |  |  |  |  |  |  |  |  |  |  |  |  |  |  |  |
| Cyprus |  |  |  |  |  |  |  |  |  |  |  |  |  |  |  |  |  |  |
| Czech Republic |  |  |  |  |  |  |  |  |  |  |  |  |  |  |  |  |  |  |
| Denmark |  |  |  |  |  |  |  |  |  |  |  |  |  |  |  |  |  |  |
| Estonia |  |  |  |  |  |  |  |  |  |  |  |  |  |  |  |  |  |  |
| Finland |  |  |  |  |  |  |  |  |  |  |  |  |  |  |  |  |  |  |
| France |  |  |  |  |  |  |  |  |  |  |  |  |  |  |  |  |  |  |
| Germany |  |  |  |  |  |  |  |  |  |  |  |  |  |  |  |  |  |  |
| Greece |  |  |  |  |  |  |  |  |  |  |  |  |  |  |  |  |  |  |
| Hungary |  |  |  |  |  |  |  |  |  |  |  |  |  |  |  |  |  |  |
| Ireland |  |  |  |  |  |  |  |  |  |  |  |  |  |  |  |  |  |  |
| Italy |  |  |  |  |  |  |  |  |  |  |  |  |  |  |  |  |  |  |
| Latvia |  |  |  |  |  |  |  |  |  |  |  |  |  |  |  |  |  |  |
| Lithuania |  |  |  |  |  |  |  |  |  |  |  |  |  |  |  |  |  |  |
| Luxembourg |  |  |  |  |  |  |  |  |  |  |  |  |  |  |  |  |  |  |
| Malta |  |  |  |  |  |  |  |  |  |  |  |  |  |  |  |  |  |  |
| Netherlands |  |  |  |  |  |  |  |  |  |  |  |  |  |  |  |  |  |  |
| Poland |  |  |  |  |  |  |  |  |  |  |  |  |  |  |  |  |  |  |
| Portugal |  |  |  |  |  |  |  |  |  |  |  |  |  |  |  |  |  |  |
| Romania |  |  |  |  |  |  |  |  |  |  |  |  |  |  |  |  |  |  |
| Slovakia |  |  |  |  |  |  |  |  |  |  |  |  |  |  |  |  |  |  |
| Slovenia |  |  |  |  |  |  |  |  |  |  |  |  |  |  |  |  |  |  |
| Spain |  |  |  |  |  |  |  |  |  |  |  |  |  |  |  |  |  |  |
| Sweden |  |  |  |  |  |  |  |  |  |  |  |  |  |  |  |  |  |  |

Abbreviations: ATMP, advanced therapy medicinal product; MS, Member State.

1. **Supplementary Figures**

**
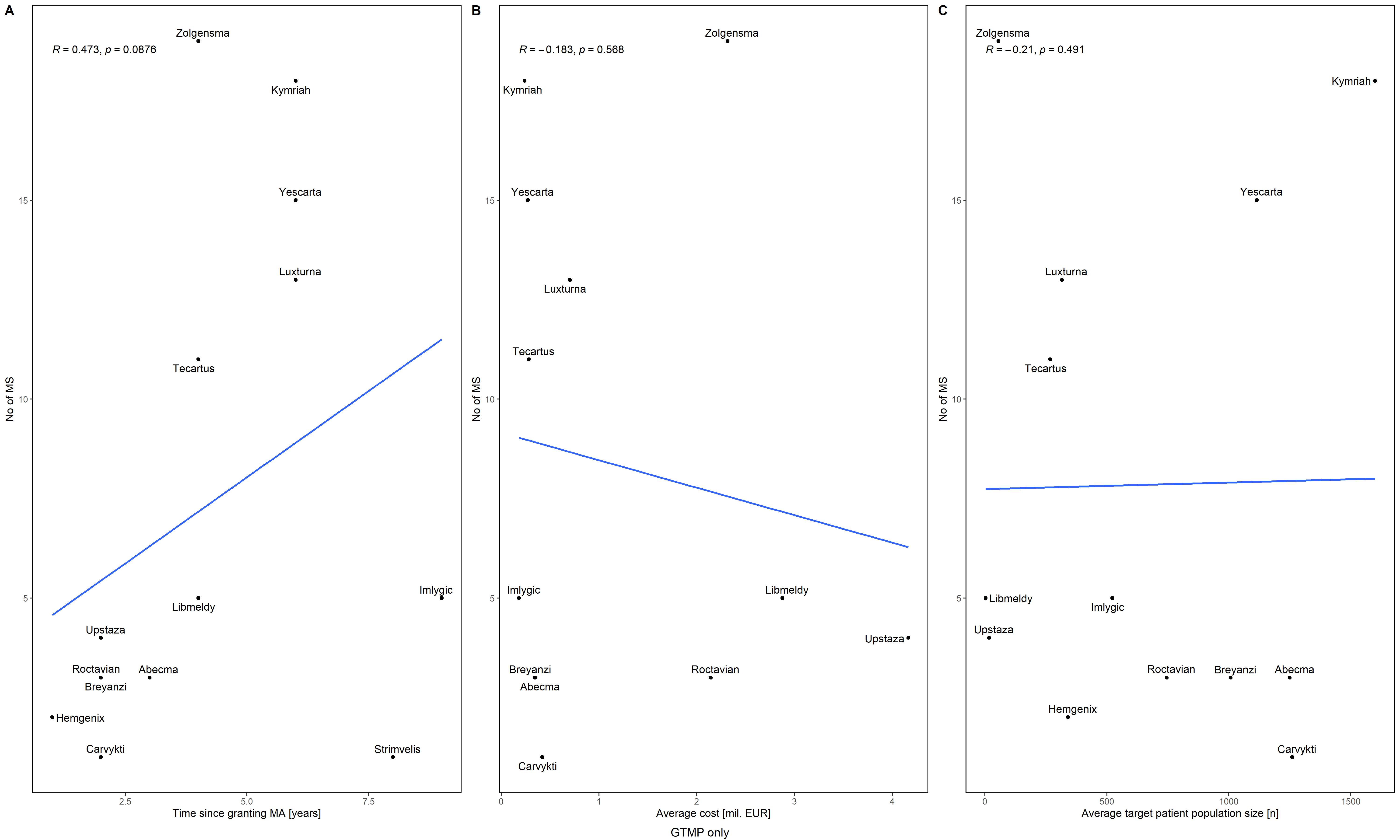
**

**Supplementary Figure 1.** Relation between gene therapy medicinal products (GTMP) availability in EU Member States and (A) time since granting marketing authorisation, (B) cost, (C) size of the target patient population.

Abbreviations: GTMP, gene therapy medicinal products; MA, marketing authorisation; MS, Member States


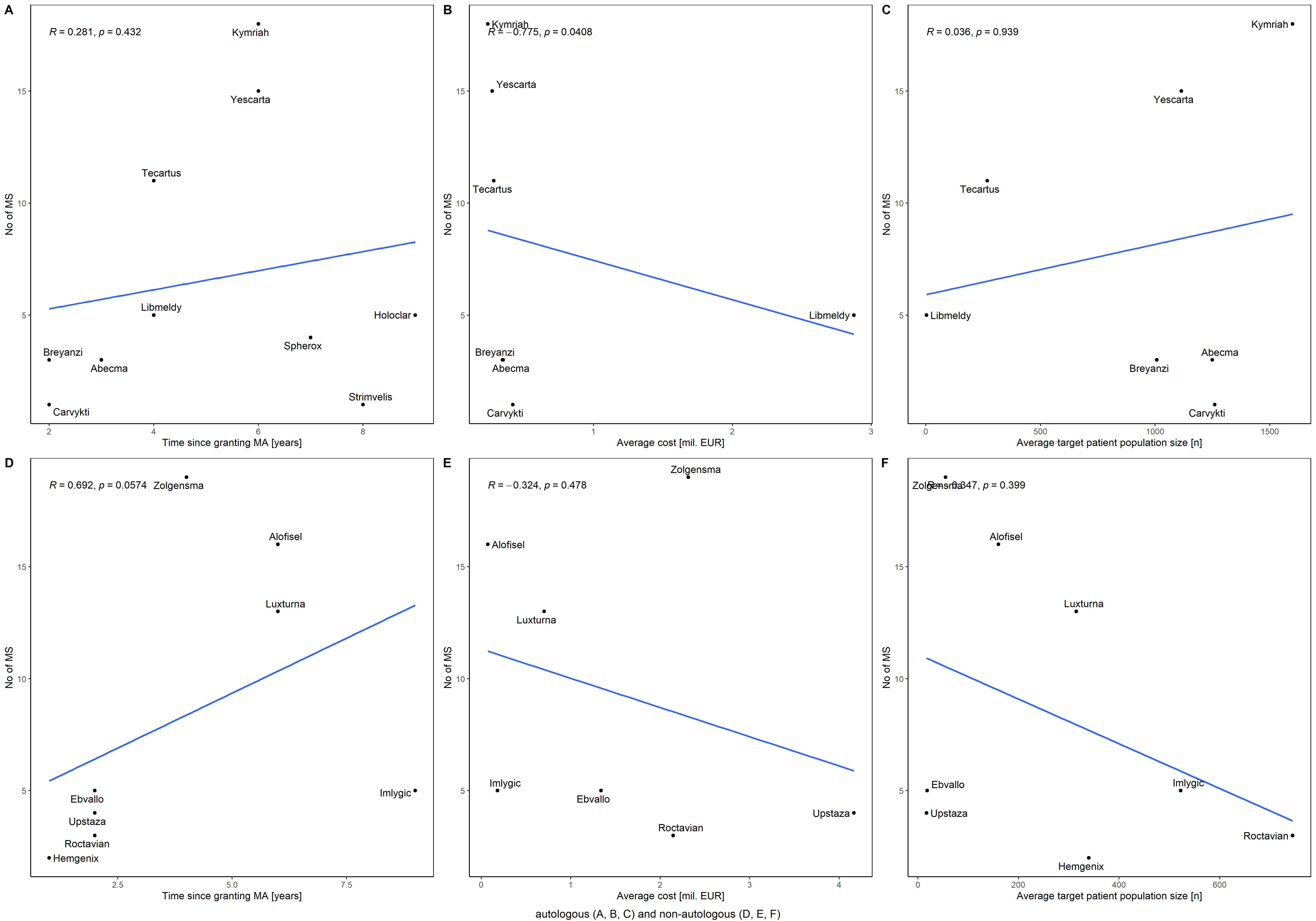


**Supplementary Figure 2.** Relation between ATMP availability in the Member States and (A) time since granting marketing authorisation, (B) cost, (C) size of the target patient population for autologous products; (D) time since granting marketing authorisation, (E) cost, (F) size of the target patient population for non-autologous products.

Abbreviations: ATMP, advanced therapy medicinal products; MA, marketing authorisation; MS, Member States


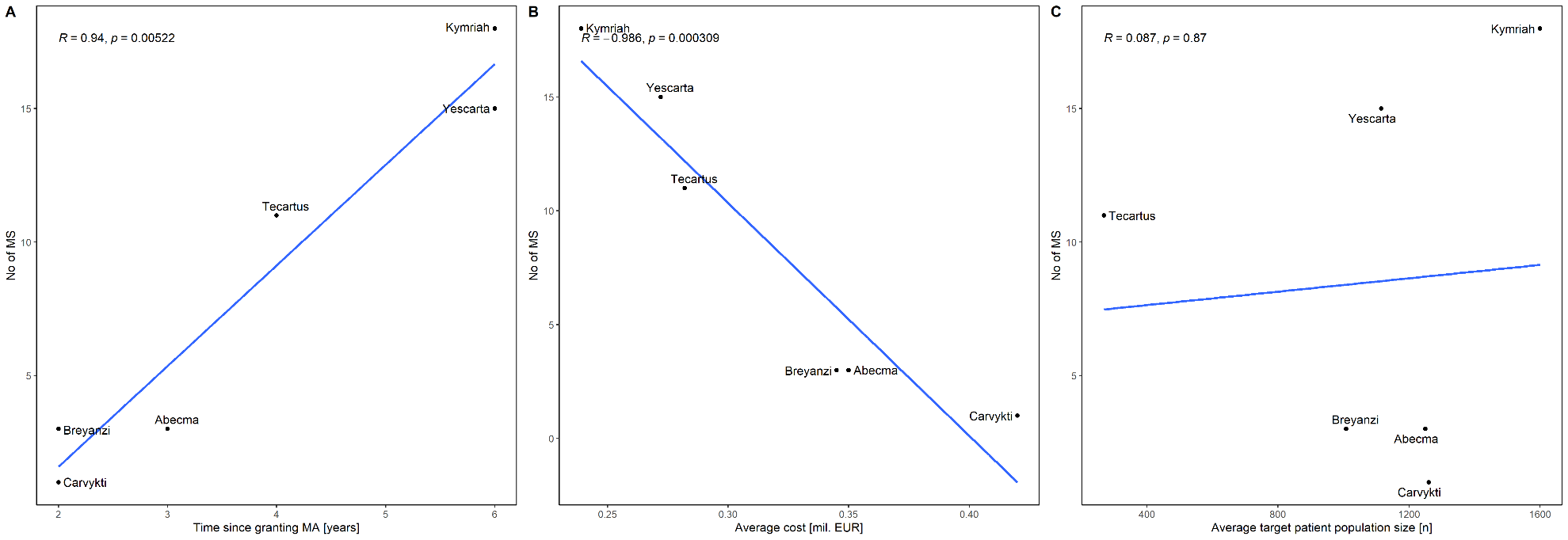


**Supplementary Figure 3.** Relation between CAR T-cell therapies availability in the Member States and (A) time since marketing granting authorisation, (B) cost, (C) size of the target patient population.

Abbreviations: CAR, chimeric antigenic receptor; MA, marketing authorisation; MS, Member States

1. **Supplementary References**
2. Bundesamt für Sicherheit im Gesundheitswesen. Arzneispezialitätenregister. <https://aspregister.basg.gv.at/aspregister/faces/aspregister.jspx>. Accessed 5 March 2024.
3. Österreichische Sozailversicherung. Der Erstattungskodex (pdf). <https://www.sozialversicherung.at/cdscontent/?contentid=10007.844497&portal=svportal>. Accessed 20 February 2024.
4. PharmaStatus. The online application on the availability of medicines. <https://pharmastatus.be/>. Accessed 5 March 2024.
5. Rijksinstituut voor ziekte- en invaliditeitsverzekering. Zoekmodules SSP - bijgewerkt op. <https://webappsa.riziv-inami.fgov.be/ssp/ProductSearch>. Accessed 20 February 2024.
6. Bulgarian Drug Agency. Register of pharmaceutical products. <https://www.bda.bg/en/registers/register-of-pharmaceutical-products>. Accessed 20 February 2024.
7. National Council on Prices and Reimbursement of Medicinal Products. Registers of National Council on Prices and Reimbursement of Medicinal Products. <https://portal.ncpr.bg/registers/pages/register/list-medicament.xhtml>. Accessed 20 February 2024.
8. National Health Insurance Fund. 2024 – Списъци с лекарствени продукти. <https://www.nhif.bg/bg/medicine_food/medical-list/2024>. Accessed 20 February 2024.
9. HALMED. Medicinal Products Database. <https://www.halmed.hr/en/Lijekovi/Baza-lijekova/>. Accessed 5 March 2024.
10. HZZO. Objavljene liste lijekova. <https://hzzo.hr/zdravstvena-zastita/objavljene-liste-lijekova>. Accessed 21 February 2024.
11. Pharmaceutical Services. Price list of Medicinal Products. <https://www.moh.gov.cy/Moh/phs/phs.nsf/pricelist_en/pricelist_en?opendocument>. Accessed 21 February 2024.
12. Pharmaceutical Services. Product search. <https://www.phs.moh.gov.cy/human-search/home.xhtml>. Accessed 5 March 2024.
13. Státní ústav pro kontrolu léčiv. Opatření obecné povahy. <https://sukl.gov.cz/prumysl/leciva/ceny-a-uhrady/opatreni-obecne-povahy/zverejnena-oop/>. Accessed 5 March 2024.
14. Státní ústav pro kontrolu léčiv. Přehled správních řízení. <https://sukl.gov.cz/modules/procedures/>. Accessed 5 March 2024.
15. Státní ústav pro kontrolu léčiv. Otevřená data. <https://opendata.sukl.cz/?q=katalog-otevrenych-dat>. Accessed 5 March 2024.
16. Danish Medicine Agency. Forside – www.medicinpriser.dk. <https://www.medicinpriser.dk/>. Accessed 5 March 2024.
17. Medicinrådet. Anbefalinger – nye lægemidler og indikations-udvidelser. <https://medicinraadet.dk/anbefalinger-og-vejledninger/laegemidler-og-indikationsudvidelser>. Accessed 5 March 2024.
18. Republic of Estonia, Agency of Medicines. Register of Medicinal Products. <https://www.ravimiregister.ee/en/default.aspx?pv=HumRavimid.Otsing>. Accessed 5 March 2024.
19. Tervisekassa. Soodusravimid. <https://www.tervisekassa.ee/partnerile/ravimitest/soodusravimid>. Accessed 21 February 2024.
20. Fimea. FimeaWeb. <https://fimea.fi/en/databases_and_registers/fimeaweb>. Accessed 5 March 2024.
21. Kela. Medicinal Products Database. <https://asiointi.kela.fi/laakekys_app/LaakekysApplication?kieli=en>. Accessed 21 February 2024.
22. ANSM. Répertoire des Spécialités Pharmaceutiques. <https://agence-prd.ansm.sante.fr/php/ecodex/index.php>. Accessed 5 March 2024.
23. Haute Autorité de Santé. Avis et décisions sur les medicaments. <https://www.has-sante.fr/jcms/p_3281266/fr/avis-et-decisions-sur-les-medicaments>. Accessed 22 February 2024.
24. Ministére du Travail, de la Santé, des Solidarités et des Familles. Référentiel des indications des spécialités pharmaceutiques inscrites sur la liste en sus. <https://sante.gouv.fr/soins-et-maladies/medicaments/professionnels-de-sante/autorisation-de-mise-sur-le-marche/la-liste-en-sus/article/referentiel-des-indications-des-specialites-pharmaceutiques-inscrites-sur-la>. Accessed 22 February 2024.
25. Gemeinsamer Bundesausschuss. Nutzenbewertung von Arzneimitteln. <https://www.g-ba.de/bewertungsverfahren/nutzenbewertung/>. Accessed 22 February 2024.
26. CGM Lauer. Lauer-Taxe Onlinde 4.0. <https://portal.cgmlauer.cgm.com/LF/default.aspx?p=12000>. Accessed 5 March 2024.
27. Υπουργείου Υγείας. Φάρμακα Υψηλού Κόστους – καταλόγων για τη θεραπεία σοβαρών ασθενειών. <https://www.moh.gov.gr/articles/times-farmakwn/farmaka-ypshloy-kostoys>. Accessed 22 February 2024.
28. National Organization for Medicines. Product search. <https://services.eof.gr/human-search/home.xhtml?lang=en>. Accessed 5 March 2024.
29. Nemzeti Népegészségügyi és Gyógyszerészeti Központ. Gyógyszer-adatbázis. <https://ogyei.gov.hu/gyogyszeradatbazis/>. Accessed 5 March 2024.
30. Nemzeti Egészségbiztosítási Alapkezelő. Publikus Gyógyszertörzs (PUPHA) – Végleges. <https://www.neak.gov.hu/felso_menu/szakmai_oldalak/gyogyszer_segedeszkoz_gyogyfurdo_tamogatas/egeszsegugyi_vallalkozasoknak/pupha/Vegleges_PUPHA>. Accessed 5 March 2024.
31. Health Products Regulatory Authority. Find a medicine. <https://www.hpra.ie/homepage/medicines/medicines-information/find-a-medicine>. Accessed 22 February 2024.
32. HSE - PCRS. Search Reimbursable Items. <https://www.sspcrs.ie/druglist/pub>. Accessed 22 February 2024.
33. National Centre for Pharmacoeconomics. Drugs. <https://www.ncpe.ie/category/drugs/>. Accessed 22 February 2024.
34. Italian Medicines Agency. List of Class A and Class H medicinal products. <https://www.aifa.gov.it/en-US/web/guest/liste-farmaci-a-h>. Accessed 23 February 2024.
35. Italian Medicines Agency. Innovative medicinal products. <https://www.aifa.gov.it/en/farmaci-innovativi>. Accessed 23 February 2024.
36. Italian Medicines Agency. Legge 189-2012. <https://www.aifa.gov.it/en/legge-189-2012>. Accessed 23 February 2024.
37. Nacionālais veselības dienests. Iesniegtie pieteikumi medikamentu iekļaušanai KZS. <https://www.vmnvd.gov.lv/lv/iesniegtie-pieteikumi-medikamentu-ieklausanai-kzs>. Accessed 23 February 2024.
38. Nacionālais veselības dienests. Kompensējamo zāļu saraksti. <https://www.vmnvd.gov.lv/lv/kompensejamo-zalu-saraksti>. Accessed 23 February 2024.
39. State Agency of Medicines Republic of Latvia. Medicinal Product Register of Latvia. <https://dati.zva.gov.lv/zalu-registrs/en>. Accessed 5 March 2024.
40. Valstybinė vaistų kontrolės tarnyba prie LR Sveikatos Apsaugos Ministerijos. Parduotų vaistinėms ir ASPĮ vaistinių preparatų pakuočių kiekis. <https://vvkt.lrv.lt/lt/svarbi-informacija/parduotu-vaistiniu-preparatu-pakuociu-kiekiai-ir-ju-atsargos/parduotu-vaistinems-ir-aspi-vaistiniu-preparatu-pakuociu-kiekis/>. Accessed 5 March 2024.
41. Valstybinė ligonių kasa prie Sveikatos apsaugos ministerijos. Kompensuojamieji vaistai ir medicinos pagalbos priemonės. <https://ligoniukasa.lrv.lt/lt/veiklos-sritys/informacija-gyventojams/vaistai-ir-medicinos-pagalbos-priemones-mpp/>. Accessed 23 February 2024.
42. Vaistų ir medicinos pagalbos priemonių kainų paieška. <https://kainynas.vlk.lt/webapp/index.html>. Accessed 23 February 2024.
43. CNS. Liste des médicaments commercialisés – Triée par denomination. <https://cns.public.lu/fr/assure/publications/legislations/textes-coordonnes/liste-med-comm-tridenom.html>. Accessed 5 March 2024.
44. Medicines Authority. Advanced Search. <https://medicinesauthority.gov.mt/advanced-search>. Accessed 5 March 2024.
45. Directorate for Pharmaceutical Affairs. The Government Formulary List. <https://pharmaceuticalaffairs.gov.mt/en/resources/the-government-formulary-list/>. Accessed 5 March 2024.
46. Zorginstituut Nederland. Horizonscan geneesmiddelen. <https://www.horizonscangeneesmiddelen.nl/?lang=en>. Accessed 23 February 2024.
47. Medicijnkosten.nl. Zoeken. <https://www.medicijnkosten.nl/zoeken>. Accessed 5 March 2024.
48. Zorginstituut Nederland. Overzicht geneesmiddelen in de sluis. <https://www.zorginstituutnederland.nl/over-ons/programmas-en-samenwerkingsverbanden/horizonscan-geneesmiddelen/sluis-voor-dure-geneesmiddelen/overzicht-geneesmiddelen-in-de-sluis>. Accessed 23 February 2024.
49. Medicines Evaluation Board. Home | Medicines Information Bank. <https://www.geneesmiddeleninformatiebank.nl/ords/f?p=111:1:0:::1:P0_DOMAIN,P0_LANG:H,EN>. Accessed 23 February 2024.
50. Centrum e-Zdrowia. Zintegrowany System Monitorowania Obrotu Produktami Leczniczymi. <https://ezdrowie.gov.pl/portal/home/systemy-it/zintegrowany-system-monitorowania-obrotu-produktami-leczniczymi>. Accessed 24 February 2024.
51. KtoMaLek.pl. Wskaz leki. <https://ktomalek.pl/>. Accessed 24 February 2024.
52. Ministerstwo Zdrowia. Leki refundowane. <https://www.gov.pl/web/zdrowie/leki-refundowane>. Accessed 5 March 2024.
53. Rejestry e-Zdrowia. RPL. <https://rejestry.ezdrowie.gov.pl/rpl/search/public>. Accessed 24 February 2024.
54. Infomed. Human medicinal products database. <https://extranet.infarmed.pt/INFOMED-fo/pesquisa-avancada.xhtml>. Accessed 5March 2024.
55. INFARMED, IP. Relatórios de avaliação de financiamento público. <https://www.infarmed.pt/web/infarmed/relatorios-de-avaliacao-de-financiamento-publico>. Accessed 24 February 2024.
56. Casa Națională de Asigurări de Sănătate. Lista interactivă a medicamentelor. <https://cnas.ro/lista-medicamente/>. Accessed 25 February 2024.
57. Ministerul Sănătăţii. Catalogul Public național al prețurilor maximale ale medicamentelor de uz uman. <https://www.ms.ro/en/ministry/structure/directia-politica-medicamentului-si-a-dispozitivelor-medicale/preturi-medicamente/catalogul-public-national-al-preturilor-maximale-ale-medicamentelor-de-uz-uman/>. Accessed 25 February 2024.
58. ANMDMR - Nomenclatorul medicamentelor pentru uz uman. Lista medicamentelor din NOMENCLATOR. <https://nomenclator.anm.ro/medicamente>. Accessed 5 March 2024.
59. Ministerstvo zdravotníctva Slovenskej republiky. Zoznam kategorizovaných liekov. <https://www.health.gov.sk/?zoznam-kategorizovanych-liekov>. Accessed 25 February 2024.
60. Ministerstvo zdravotníctva Slovenskej republiky. Zoznam liekov s úradne určenou cenou 1.3.2024 – 31.3.2024. <https://www.health.gov.sk/Clanok?zuuc-202403-lieky>. Accessed 25 February 2024.
61. Národné centrum zdravotníckych informácií. Datasety spotreby humánnych liekov v Slovenskej republike. <https://www.nczisk.sk/Statisticke_vystupy/Tematicke_statisticke_vystupy/TOP-50-liekov/Spotreba_humannych_liekov_zdravotnickych_pomocok_dietetickych_potravin_SR/Pages/Datasety-spotreby-humannych-liekov-v-Slovenskej-republike.aspx>. Accessed 5 March 2024.
62. Štátny ústav pre kontrolu liečiv. Hlásenie o prerušení. <https://portal.sukl.sk/PreruseniePublic/?act=PrerusenieOznList&mId=2>. Accessed 5 March 2024.
63. JAZMP. Medicinal products authorized in Europe by now. <https://www.jazmp.si/en/human-medicines/advanced-medicinal-products-atmp/medicinal-products-authorized-in-europe-by-now/>. Accessed 26 February 2024.
64. JAZMP. List of regulated prices. <https://www.jazmp.si/en/human-medicines/pricing-of-medicinal-products/list-of-regulated-prices/>. Accessed 26 February 2024.
65. Portal za izvajalce. Podatki o porabi zdravil. <https://partner.zzzs.si/zdravila-in-zivila-za-posebne-zdravstvene-namene/podatki-o-porabi-zdravil/>. Accessed 26 February 2024.
66. Centralna baza zdravil 2. Iskanje. <http://www.cbz.si/cbz/bazazdr2.nsf/Search/$searchForm?SearchView>. Accessed 5 March 2024.
67. Ministerio de Sanidad – Profesionales de la Salud. Buscador situación financiación medicamentos. <https://www.sanidad.gob.es/profesionales/medicamentos.do>. Accessed 26 February 2024.
68. CIMA. Centro de información de medicamentos. <https://cima.aemps.es/cima/publico/home.html>. Accessed 5 March 2024.
69. VARA. Sök läkemedel. <https://vara.ehalsomyndigheten.se/vara-web/>. Accessed 26 February 2024.
70. FASS PATIENT. Startsida → För dig som är patient eller närstående. <https://www.fass.se/LIF/startpage?userType=2>. Accessed 5 March 2024.
71. Tandvårds- och läkemedelsförmånsverket TLV. Sök priser och beslut i database. <https://www.tlv.se/beslut/sok-priser-och-beslut-i-databasen.html>. Accessed 26 February 2024.
